# Supplementary material for: Cambial Age Influences PCD Gene Expression during Xylem Development and Heartwood Formation
Source: Plants (Basel). 2023 Dec 4;12(23):4072. doi: 10.3390/plants12234072 (PMC10707818; doi:10.3390/plants12234072)
Supplement: Supplementary file 1 [file plants-12-04072-s001.zip › plants-2717502_Supplementary figure S1.pdf]

plants

Supplementary Material

Cambial Age Influences PCD Gene Expression during Xylem Development and Heartwood Formation

Yulia L. Moshchenskaya *, Natalia A. Galibina, Tatiana V. Tarelkina, Ksenia M. Nikerova, Maksim A. Korzhenevsky and Ludmila I. Semenova

Forest Research Institute, Karelian Research Centre of the Russian Academy of Sciences, 11 Pushkinskaya St., 185910 Petrozavodsk, Russia; karelt@mail.ru (T.V.T.); knikerova@yandex.ru (K.M.N.); maksim.korjan@gmail.com (M.A.K.); mi7enova@gmail.com (L.I.S.)
* Correspondence: tselishcheva.yulia@mail.ru; Tel.: +7-(8142)-768160

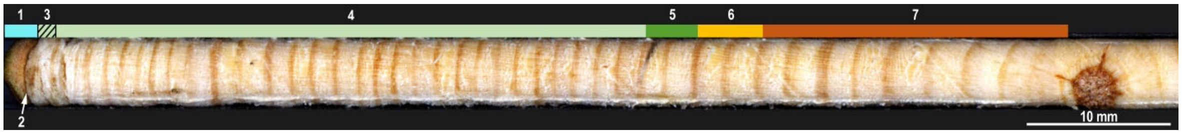

Supplementary Figure S1. A photograph of a core showing the location of the stem tissues selected for analysis. The numbers indicate the following: 1 – bark, 2 – cambium, 3 – outer sapwood, 4 – sapwood, 5 - inner sapwood, 6 – transition zone, 7 – heartwood.
