# Supplementary material for: Cambial Age Influences PCD Gene Expression during Xylem Development and Heartwood Formation
Source: Plants (Basel). 2023 Dec 4;12(23):4072. doi: 10.3390/plants12234072 (PMC10707818; doi:10.3390/plants12234072)
Supplement: Supplementary file 1 [file plants-12-04072-s001.zip › plants-2717502_Supplementary figure S2.pdf]

plants

Supplementary Material

Cambial Age Influences PCD Gene Expression during Xylem Development and Heartwood Formation

Yulia L. Moshchenskaya *, Natalia A. Galibina, Tatiana V. Tarelkina, Ksenia M. Nikerova, Maksim A. Korzhenevsky and Ludmila I. Semenova

Forest Research Institute, Karelian Research Centre of the Russian Academy of Sciences, 11 Pushkinskaya St., 185910 Petrozavodsk, Russia; karelt@mail.ru (T.V.T.); knikerova@yandex.ru (K.M.N.); maksim.korjan@gmail.com (M.A.K.); mi7enova@gmail.com (L.I.S.)

* Correspondence: tselishcheva.yulia@mail.ru; Tel.: +7-(8142)-768160

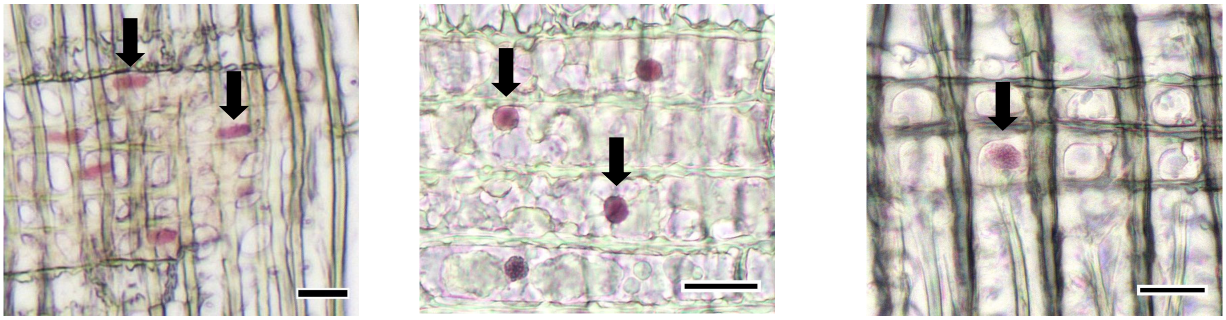

(**a**) (**b**) (**c**)

Supplementary Figure S2. Radial sections of pine xylem. (**a**) Outer sapwood. The nuclei in the ray parenchyma cells are oblong-elliptical (black arrow). (**b**) Inner sapwood. The nuclei in the ray parenchyma cells display evident pycnotic degeneration. They are rounded due to water loss and chromatin condensation (black arrows). (**c**) Transition zone. The destruction of the nuclear membranes occurs. Chromatin stained with acetocarmine can be observed in cells (black arrow). The scale bars are 25 μm.
